# Methylation alterations are not a major cause of PTTG1 missregulation

**DOI:** 10.1186/1471-2407-8-110

**Published:** 2008-04-21

**Authors:** Manuel Hidalgo, Jose Jorge Galan, Carmen Sáez, Eduardo Ferrero, Carolina Castilla, Reposo Ramirez-Lorca, Pablo Pelaez, Agustin Ruiz, Miguel A Japón, Jose Luis Royo

**Affiliations:** 1 Service of General Surgery-B, Hospital Universitario 12 de Octubre, Madrid, 28041, Spain; 2 Department of Structural Genomics, Neocodex SL, Avda Charles Darwin 6 Acc A. Seville, 41092, Spain; 3 Department of Pathology, Hospital Universitario Virgen del Rocío, Seville, 41013, Spain

## Abstract

**Background:**

On its physiological cellular context, PTTG1 controls sister chromatid segregation during mitosis. Within its crosstalk to the cellular arrest machinery, relies a checkpoint of integrity for which gained the over name of *securin*. PTTG1 was found to promote malignant transformation in 3T3 fibroblasts, and further found to be overexpressed in different tumor types. More recently, PTTG1 has been also related to different processes such as DNA repair and found to trans-activate different cellular pathways involving c-myc, bax or p53, among others. PTTG1 over-expression has been correlated to a worse prognosis in thyroid, lung, colorectal cancer patients, and it can not be excluded that this effect may also occur in other tumor types. Despite the clinical relevance and the increasing molecular characterization of PTTG1, the reason for its up-regulation remains unclear.

**Method:**

We analysed PTTG1 differential expression in PC-3, DU-145 and LNCaP tumor cell lines, cultured in the presence of the methyl-transferase inhibitor 5-Aza-2'-deoxycytidine. We also tested whether the CpG island mapping *PTTG1 *proximal promoter evidenced a differential methylation pattern in differentiated thyroid cancer biopsies concordant to their PTTG1 immunohistochemistry status. Finally, we performed whole-genome LOH studies using Affymetix 50 K microarray technology and FRET analysis to search for allelic imbalances comprising the *PTTG1 *locus.

**Conclusion:**

Our data suggest that neither methylation alterations nor LOH are involved in PTTG1 over-expression. These data, together with those previously reported, point towards a post-transcriptional level of missregulation associated to PTTG1 over-expression.

## Background

Human pituitary tumor-transforming protein (PTTG1) is a 22-kDa protein proven to be tumorigenic in NIH3T3 fibroblasts [1] and further abundantly expressed in many tumors. Under physiological conditions, PTTG1 expression is found to be regulated through the cell cycle, with a peak at G2/M phase. PTTG1 primary function is related to the control of sister chromatid separation to the opposite spindle poles. According to this activity, genomic imbalance as a result of chromosome missegregation is a rationale for the oncogenic potential of upregulated PTTG1 expression. In fact, PTTG1 over-expression has been associated with aneuploidy generation, what correlates with a differentiated prognosis in multiple tumor types [2,3]. In addition, PTTG1 and Fibroblast Growth Factor (FGF) together form a positive feedback loop and stimulate tumor angiogenesis [4,5]. Besides, PTTG1 may play a role in double strand break reparation trough Ku-70 and regulating cell proliferation and apoptosis transactivating *c-myc *and *bax *[6-8]. Thus, there may be several possible mechanisms for PTTG1 tumorigenesis [8].

From the pathological point of view, PTTG1 has been found to be expressed at high levels in human pituitary adenomas and other malignant tumors including breast, lung, prostate, ovary and thyroid cancer, as well as in haematopoietic neoplasias [9-15]. PTTG1 expression has been correlated with lymph node invasion in colorectal cancer and was proposed as an independent prognostic molecular biomarker [16]. Moreover, increased PTTG1 expression levels and early tumor recurrence has been found in different cancer series [11,17]. Finally, we have recently reported that PTTG1 is highly expressed in two thirds (65%) of the differentiated thyroid cancers of Spanish origin, and was shown to be an independent prognostic factor for persistent disease among DTC patients [15].

Despite the large amount of data available, the molecular mechanisms underlying PTTG1 over-expression have not been clarified so far. In a previous work, Kanakis and coworkers performed a sequencing scan in sixteen tumor biopsies from pituitary adenoma patients, searching for small deletion/insertion within those regions previously identified to be controlling PTTG1 expression [18]. In their study they conclude that promoter mutations do not play a mayor role for the enhanced PTTG1 transcription and suggested that promoter hypomethylation may be responsible for PTTG1 over-expression.

It has been proposed that demethylation along the genome largely affects the intergenic and intronic regions of DNA, and it is believed to result in chromosomal instability and increased mutation events [19]. Under this hypothesis, a CpG island identified close to the core *PTTG1 *promoter may display a differential methylation pattern in normal tissues when compared to their corresponding tumors, and be also different between those tumors with different PTTG1 expression levels. On the other hand, other structural events such as gene amplification could also explain the different over-expression levels present in both tumor biopsies and tumor cell lines. Here, we first investigate whether epigenetic and structural alterations may explain PTTG1 upregulation in both tumor cell lines and thyroid cancer biopsies. We have studied the methylation status in a CpG island characterized in the proximal promoter region of *PTTG1*, using methylation-specific PCRs (MSPs). In addition, to test the presence of a putative epigenetic control over PTTG1 expression we performed PTTG1 expression analysis in three tumor cell lines with different basal expression levels. We also evaluated the possibility that loss of heterozygosity (LOH) events involving the *PTTG1 *locus could also explain the higher amount of protein present in different tumor biopsies and tumor cell lines.

## Methods

### Patients and controls

To isolate germline DNA, we obtained 5 ml of peripheral blood. DNA extraction was performed automatically according to standard procedures using Magnapure DNA isolation system (Roche, Mannheim, Germany). Thyroid biopsies were from 47 paraffin-embedded tissues (FPET) of unrelated individuals with differentiated thyroid carcinoma (DTC) and matched adjacent unaffected tissues from the same individual. DNA extraction from FPET was performed as previously described [20]. DNA samples from 185 controls from Spanish population were obtained from a previous work [21]. Institutional and ethical review board from the referral centres approved the consecution of this project

### Immunohistochemical analysis of PTTG1

Immunohistochemistry was performed as previously described [15]. Paraffin tissue sections (5 μm thick) were dewaxed in xylene and rehydrated in a series of graded alcohols. Sections were immersed in 3% H_2_O_2 _aqueous solution for 30 min to quench endogenous peroxidase activity, and then covered with 10% normal serum in Tris-buffered saline to block nonspecific binding sites. A rabbit polyclonal anti-PTTG1 antibody was available for this study. Specificity control experiments for this antibody have been described previously [13,22]. Sections were incubated overnight with a 1:500 dilution of primary anti-PTTG1 antibody. After several washes in Tris buffer, peroxidase-labeled secondary antibodies and 3,3'-diaminobenzidine were applied to develop immunoreactivity, according to manufacturer's protocol (EnVision; Dako, Glostrup, Denmark). The slides were then counterstained with hematoxylin and mounted in DPX (BDH Laboratories, Poole, UK). Sections in which primary antibody was omitted were used as negative controls.

### Methylation-specific PCR

Genomic sequence from *PTTG1 *was obtained from public databases (see Availability and requirements section for URL, Unigene Hs.350966) and was analyzed using the freely available CpG Island Searcher (see Availability and requirements section for URL) developed according to the algorithms previously reported [23] and confirmed using the sequence analysis tool from the European Bioinformatic Institute (see Availability and requirements section for URL). Genomic DNA from both tumor samples and their corresponding healthy tissues were treated with bisulfite sulfoxide for 6 h at 50°C and further purified using Methyldetector Kit (Active Motif, Carlsbad, California, USA) according to manufacturer's instructions.

### Cell cultures

Human prostate cancer cell lines PC-3, DU-145 and LNCaP were obtained from the Cell Line Collection (Interlab, Genoa, Italy) and routinely grown in RPMI 1640 supplemented with 10% heat-inactivated fetal bovine serum, 100 U/ml penicillin, 100 μg/ml streptomycin and 1 mM glutamine in a 37°C, humidified incubator under 5% CO_2_. 5-Aza-2'-deoxycytidine (5-Aza) (Sigma, St. Louis, Missouri, USA) was prepared in dimethyl sulfoxide (DMSO). 3 × 10^5 ^cells were plated in 25 cm^2 ^culture flasks and allowed to adhere for 24 h. Then the cultures were treated either with 2 μM 5-Aza or DMSO for 78 h, with a medium and drug change every 24 h. Cell cultures were harvested by trypsinization.

### Western blotting

Western blotting was performed essentially as previously described [24]. Briefly, twenty micrograms of total protein, as determined by using BCA protein assay kit (Pierce, Rockford, Illinois, USA), were separated by SDS-PAGE on 4–20% gradient polyacrylamide gels (Invitrogen, Carlsbad, California, USA). Gels were electroblotted onto nitrocellulose membranes (Amersham, Little Chalfont, UK). Ponceau Red staining was used to confirm equal loading. For immunodetection, blots were blocked in 1% blocking reagent (Roche) in 0.05% Tween 20-TBS for 1 h and incubated with primary antibody overnight at 4°C diluted in blocking buffer. Blots were then washed in 0.05% Tween 20-TBS and incubated with either goat anti-mouse (1:20,000; Amersham) or goat anti-rabbit (1:20,000; Amersham) peroxidase-labeled antibodies in blocking buffer for 1 h. Enhanced chemoluminescent system was applied according to the manufacturer's protocol (Amersham). Monoclonal anti-Caspase 7 antibody was available from BD Biosciences (San Jose, California, USA). Dilutions used in Western blots were anti-PTTG1 (1:1,000), anti-Caspase 7 (1:2,000) and β-tubulin (1:500).

### Reverse-transcription and quantitative PCR

Total RNA was extracted using the Purescript^® ^RNA Isolation Kit (Gentra, Minneapolis, USA) according to the manufacturer's protocol. 0.5 μg of total RNA was subjected to DNase I (Invitrogen) digestion and subsequently processed to cDNA by reverse transcription with Transcriptor First Strand cDNA Synthesis Kit (Roche) according to the manufacturer's protocol. All PCR reactions were performed in a 25 μl reaction volume on the SmartCycler II Real-Time PCR Detection System (Cepheid, Sunnyvale, California, USA) using the Platinum SYBR Green qPCR SuperMix-UDG (Invitrogen) and 500 nM of each specific primer. PCR efficiencies were calculated using a relative standard curve derived from 10-fold dilution series of pooled cDNA mixture (a 10-fold dilution series with four measuring points). The levels of PTTG1 and the housekeeping gene RPL13A in each sample were quantified by measuring the Ct values in duplicate. These mean Ct values were transformed to quantities using the delta-Ct method. The sample with the lowest value was assigned the value 1. The quantity of PTTG1 transcript was divided by the quantity of RPL13A to obtain a normalized value.

### Affymetrix 50 K microarrays

Affymetrix (San Diego, California, USA) 50 K Xba I arrays were performed according to manufacturer's instructions. Briefly, 250 ng of high molecular weight genomic DNAs were digested with Xba I for 2 h. Next, a double-stranded DNA adaptor was added and ligated to cohesive Xba I ends. Following, PCR was performed in order to reduce DNA complexity, being amplified those segments below 5 Kb. PCR products were purified and digested using DNAse I to generate fragments of aprox. 200 bp, that were subsequently biotinylated. Samples were hybridized at 48°C for 16 h and washed and stained using Affymetrix fluidic station 450. Microchips were scanned the same day using an Affymetrix 7G GeneChip scanner and analysed using CNAT 4.0 software.

### Genotyping using fluorescence resonance energy transfer (FRET)

Two informative polymorphisms mapping *PTTG1 *gene were selected from HapMap database (rs3811999, Minor Alelle Frequency (MAF): 0.4 and rs4921281, MAF: 0.21). Every PCR reaction was performed in a final volume of 20 μl, containing 25 ng of genomic DNA, and 1× buffer from Light Cycler^® ^Molecular probe master mix (Roche), 5 pmol of each primer and 2 pmol of each genotyping probe. PCRs were performed in a Light Cycler^® ^480. Primers and amplification conditions are described in table 1. Upon amplification, a melting curve from 50°C to 80°C was performed while recording the Cy5 fluorescence.

**Table 1:** Primers and amplification conditions.

**Primer**	**Sequence (5'-3')**	**Amplicon**	**Amplification conditions**
PTTG MF1	TTTCGGATTGTTAATTGGATTAAC	168 bp	1. [95°C for 7'] 2. [95°C for 0'', 60°C for 20'', 72°C for 25 '']x45 3. Melting curve
PTTG MR1	AAAAACAAAAACTAAACAACGAA		2. [95°C for 0'', 60°C for 20'', 72°C for 25 ''] × 45
	
PTTG UF1	TTGGATTGTTAATTGGATTAATGG	166 bp	3. Melting curve
PTTG UR1	AAAAACAAAAACTAAACAACAAA		

PTTG MF2	GTTTTATTTGGTGATTACGTTTACG	123 bp	1. [95°C for 7']
PTTG MR2	ACCGCATTCATCTAAAACCG		2. [95°C for 0'', 60°C for 20'', 72°C for 25 ''] × 45
	
PTTG UF2	TTTTATTTGGTGATTATGTTTATGG	125 bp	3. Melting curve
PTTG UR2	ACAACCACATTCATCTAAAACCAC		

PTTG-1F	AAAGTAGCTACCATTCCTGC	124 bp	1. [95°C for 7'] 2. [95°C for 30'', 60°C for 30'', 72°C for 30 '']x45 3. [72°C for 3']
PTTG-1R	TGCCCTGTAAAAGCAAAAT		2. [95°C for 30'', 60°C for 30'', 72°C for 30 ''] × 45
PTTG-1 Anc	FL-AATAGCCCAACATAATAGAATCTATTTT-Ph		3. [72°C for 3']
PTTG-1 Sen	GCTACCATTCCTGCCTTAATA-Cy5		

PTTG-3F	TGCTGACAGGTGCTGGTACT	128 bp	1. [95°C for 7'] 2. [95°C for 30'', 60°C for 30'', 72°C for 30 '']x45 3. [72°C for 3']
PTTG-3R	AAGAAGCCATAATCCTTAGTTTTCA		2. [95°C for 30'', 60°C for 30'', 72°C for 30 ''] × 45
PTTG-3 Anc	FL-TTTGAGTCATGCTACTCGAATTACAT-Ph		3. [72°C for 3']
PTTG-3 Sen	GGTACTTAAATTTCCGATTTTAAC-Cy5		

p16 Forward outer	GTAGGTGGGGAGGAGTTTAGTT	283 bp	1. [94°C for 3']
p16 Reverse outer	TCTAATAACCAACCAACCCCTCC		2. [94°C for 30'', 50°C for 30'', 72°C for 30''] × 20 3.[72°C for 4']

p16 Forward inner	GGGGGAGATTTAATTTGG	190 bp	1. [94°C for 3'] 2. [94°C for 30'', 50°C for 30'', 72°C for 30''] × 30 3. [72°C for 4']
p16 Reverse inner	CCCTCCTCTTTCTTCCTC		2. [94°C for 30'', 50°C for 30'', 72°C for 30''] × 30 3. [72°C for 4']

qPCR PTTG Fw	AGGCACCCGTGTGGTTGCT	126 bp	1. [50°C for 2']
qPCR PTTG Rev	TAAGGCTGGTGGGGCATC		2. [95°C for 2']
	
qPCR RPL13A Fw	CCTGGAGGAGAAGAGGAAAGAGA	125 bp	3. [95°C for 5'', 60°C for 30''] × 40
qPCR RPL13A Rev	TTGAGGACCTCTGTGTATTTGTCAA		4. Melting curve

## Results

### Epigenetic regulation of PTTG1 expression

**Figure 1:**
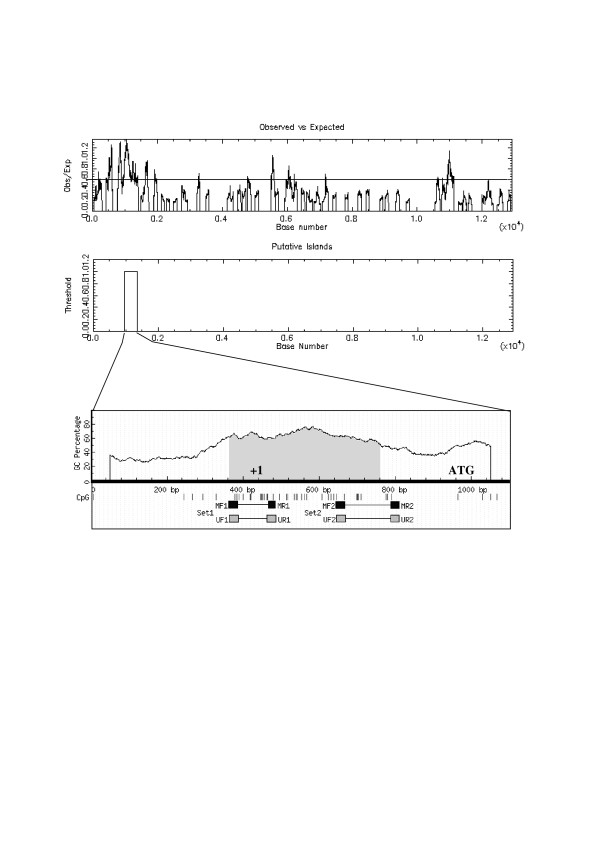
**Structural search for CpG islands within *PTTG1***. Design of the primer sets for MSPs. The upper graph represents the GC content expressed as observed/expected along the entire *PTTG1 *genomic sequence according to the CpG Island Searcher. The middle graph represents the region identified as a putatively functional CpG island, mapping the proximal promoter. This region was subjected to further analysis to refine the critical region and design the necessary MSP primer sets. In the lower graph the critical region is designated as a shadow below the CG percentage. Vertical bars represent each CG pair. Squares represent the different primer sets, designed to amplify if methylated before treatment (black) or unmethylated (grey)

**Figure 2:**
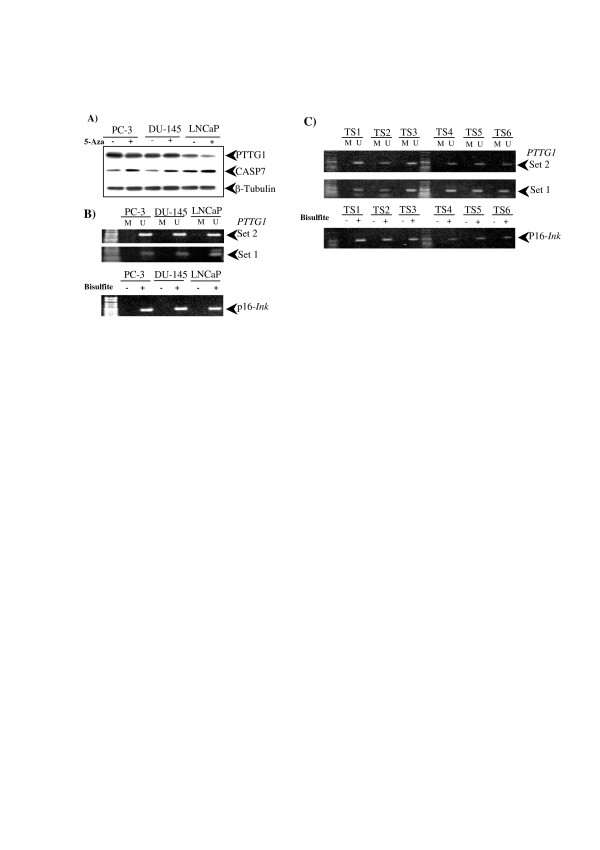
**Methylation analysis of PTTG1 expression**. A) Western blotting analysis of each cell line cultured in the presence or absence of 5-Aza. B) MSP analysis of tumor cell lines. Converted DNA is subjected to three different PCR reactions. For each primer set, two primer pairs are used to determine if the *PTTG1 *locus is methylated (M) or unmethylated (U). Bisulfite conversion control is performed by amplifying p16-*Ink *locus. C) MSP analysis of tumor samples with low (TS1-3) or high PTTG1 expression levels (TS4-6).

Genomic DNA sequence from *PTTG1 *gene was downloaded from public databases available and subjected to two different CpG island discovery softwares. Both programs identified a region mapping the 5' site of *PTTG1*, from -56 to -712 (in relation to translation start site) with a high probability of being subjected to epigenetic control (Figure 1). In order to study a putative epigenetic control over PTTG1 expression, PC-3, DU-145 and LNCaP cell lines were selected as *in vivo *models. These cell lines have been utilized for PTTG1 expression control analysis since they exhibit different PTTG1 expression levels under normal culture conditions (Figure 2A). Cells were cultured either in the presence or absence of 2 μM 5-Aza, which is known to inhibit the methyl-transferase complex, causing genome-wide demethylation. After 78 h incubation in the presence of the drug, cells were harvested and total protein was extracted and normalized. Samples were subjected to SDS-PAGE and western blot, using anti-β-tubulin as additional loading control. As positive induction control we measured CASP7 expression, which has been reported to be epigenetically silenced under normal culture conditions. As expected, we could observe a clear up-regulation of CASP7 in those samples treated with 5-Aza (+17%, +31% and +36% for PC3, DU-145 and LNCaP, respectively). On the contrary, no up-regulation was detected for PTTG1 expression in any of the three cell lines under study (Figure 2A). In fact, PTTG1 expression exhibited a slight inhibition upon treatment. This was especially evident for PC-3 and LNCaP, which exhibited 12% PTTG1 downregulation. To confirm these data, total RNA was extracted from the different cell lines under the culture conditions previously mentioned and subjected to RT-qPCR analysis. According to these assays, *PTTG1 *mRNA expression levels were lowered in all cases 3–5% upon treatment, giving additional support to the results previously obtained (data not shown).

### Methylation-specific PCR analysis of DTC biopsies and tumor cell lines

As described above, we identified a 650 bp region with high probability of being subjected to epigenetic control. Next, two sets of primers were design to test and quantify the presence of unmethylated cytosines residues among our samples. With this approach, the same converted DNA sample is subjected to two independent PCRs; the first, using two primer pairs designed towards the unmethylated cytosines, and two primer pairs designed towards the methylated residues (Figure 1). Since bisulfite treatment strongly degrades DNA, samples from both PTTG1 positive and negative biopsies were examined for degradation in low-melting agarose gels and purity (OD_260_/OD_280 _ratio) prior to conversion treatment. Next, DNA of three representative samples from each tumor type were extracted together with DNA from its adjacent healthy tissue to be taken as inner reference as well as genomic DNAs from PC-3, DU-145 and LNCaP cells. All samples (n = 15) were treated with sodium bisulfite as stated in materials and methods, in order to convert unmethylated cytosines to uracil residues. As positive conversion control, all DNA samples were tested for amplification using nested MSP designed for p16-*ink *according to manufacturer's instructions and as previously stated [25].

Upon full conversion was confirmed, converted DNA samples were subjected to MSP analysis using both sets of primers aforementioned. To determine the sensitivity of MSPs, serial ratios of converted:untreated DNAs were performed and subjected to real-time MSP to measure the amplification crossing point [see Additional file 1]. According to MSP analysis, all tumor samples displayed unmethylated CpG island regardless their PTTG1 status, as well as their respective contra-lateral healthy tissue. PC-3, DU-145 and LNCaP cell lines also exhibited unmethylated PTTG1 promoter despite their different expression pattern (Figure 2B and 2C). Altogether, our data suggest that PTTG1 expression levels do not correlate with CpG methylation status.

### Loss of heterozygosity studies within the PTTG1 locus

**Figure 3:**
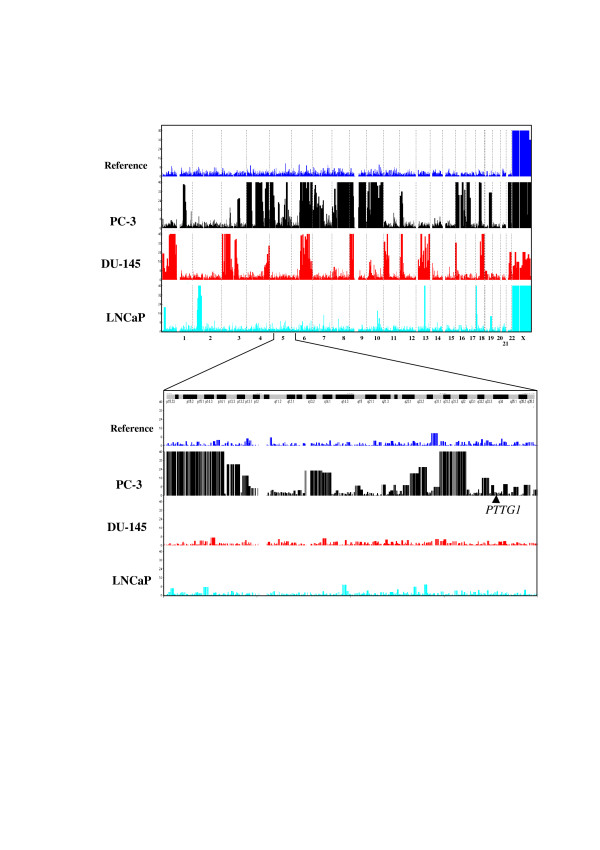
**Genome-wide LOH scan using Affymetrix 50 K *Xba *I array**. Graphs represent the LOH likelihood (Y-axis) respect to the base-pair. Chromosomes are represented in the X-axis. As a reference, we used peripheral-blood derived DNA from a healthy control from Spanish population. In the lower graph, chromosome 5 is enlarged and the *PTTG1 *locus is marked.

In order to test whether the different PTTG1 expression levels could be explained by gene amplification, we subjected PC-3, DU-145 and LNCaP cell lines and two control germline DNAs to Affymetrix 50 K Xba I array analysis. This platform offers a quantitative mapping analysis of the genome at a median intermaker distance of 16 Kb. Gene dosage and loss of heterozygosity were analysed using CNAT 4.0 software, and data from chromosome 5 was filtered and represented in figure 3. We observed that several LOH regions (threshold ≥ 15) were common to PC-3 and DU-145 cell lines, and others restricted to each one. However, none of them involved the region mapping *PTTG1 *gene.

Due to technical restrictions, the 50 K Xba I array can not be used for paraffin embedded tissues-derived DNA analysis, and after a careful bioinformatic search, no suitable microsatellite was present in our region of interest to be analyzed to detect putative LOH regions. Thus, we selected from HapMap database two highly informative polymorphisms mapping *PTTG1 *gene (rs3811999 and rs4921281) and genotyped them in a series from general Spanish population (n = 186). Both minor allele frequencies did not significantly differ from the ones described from the public database (0.37 *vs *0.4 for rs3811999 and 0.16 *vs *0.21 for and rs4921281). Thus, empirical informativeness for each SNP was 0.48 and 0.27 respectively. Following, we genotyped both the tumor samples (n = 47) and their corresponding contra-lateral healthy tissues. Perfect concordance was obtained between those genotypes obtained using tumor and healthy samples, giving additional support to Affymetrix results and supporting the theory that no LOH event had occurred involving *PTTG1 *gene.

## Discussion

Growing evidences arise from the literature linking PTTG1 over-expression to both tumor growth and a worse prognosis among independent patient series. According to a leading theory, PTTG1 over-expression may generate a chromosomal instability phenotype associated to a subset of genomic rearrangements leading to a worse prognosis. In this context, different authors proposed PTTG1 as a strong candidate for drug target design and thus, a deeper knowledge of the mechanism underlying PTTG1 upregulation in tumoral cells may contribute to the development of effective therapeutic strategies.

In this study, we have tested whether structural DNA alterations such as CpG methylation or LOH may explain PTTG1 expression levels in both cell lines and DTC tumor and healthy samples. Experiments using tumor cell lines cultured in the presence or absence of 5-Aza reveal that hypomethylation seems not to be involved in PTTG1 over-expression. On the contrary, our results may suggest that genome-wide hypomethylation obtained using 5-Aza treatment results in PTTG1 repression in three different genetic backgrounds, altough the elucidation of this point would require further investigation. According to our data derived from the MSP analysis performed over the CpG island mapping the *PTTG1 *close promoter analysed in both DTC biopsies and cell lines, the CpG island mapping *PTTG1 *close-promoter appears unmethylated in both healthy tissues and tumor samples, regardless their PTTG1 expression status.

In addition, it seems that LOH phenomena may also not be involved in PTTG1 over-expression, according to Affymetrix results and those obtained from the genotyping experiments, where no allelic imbalance has been observed. These data together with those previously reported by Kanakis et al [18] suggest the presence of other mechanism as a leading cause for PTTG1 over-expression such as post-transcriptional missregulation, leading to either a longer mRNA stabilization or increased protein mean-live. However, we can not exclude the possibility that additional epigenetic alterations such as histone acetylation or ubiquitylation may be playing important roles in regulating PTTG1 expression levels.

To date, different *PTTG1 *transcriptional regulators have been identified. Zhou et al showed that Sp1 binds together with NF-Y to *PTTG1 *promoter sequence, and that site-directed mutagenesis of the Sp1 consensus sequence resulted in approximately 70% reduction of the transcriptional activation of the promoter, whilst mutation of the NF-Y binding site resulted in 25% reduction. Deletion of both Sp1 and NF-Y sequences resulted in a 90% loss of PTTG1 promoter activity [26]. Others have identified that WldS might also be involved in PTTG1 expression control, as well as beta-catenin [27,28]. Nevertheless, no conclusive experiments have been reported to our knowledge explaining the nature of PTTG1 over-expression in tumor samples. On the other hand, if we focus on *PTTG1 *mRNA stabilization or PTTG1 degradation pathways, results are scarce. Recently, protein phosphatase 2A has been involved in the stabilization and increased mean life of PTTG1, suggesting that impaired degradation could be relevant in the upregulation of PTTG1 [29]. However, further investigation is needed to completely elucidate the underlying mechanism of PTTG1 upregulation in tumors.

## Conclusion

To date, growing evidences are being reported pointing towards a worse clinical outcome associated to PTTG1 expression. However, the mechanisms underlying such deregulation remain unclear. We failed to correlate PTTG1 expression rates with a specific methylation pattern of the CpG island mapping -56 to -712 of *PTTG1 *promoter. Moreover, culture experiments corroborate these data and suggest that genome-wide hypomethylation is not associated to PTTG1 over-expression. In addition, LOH studies were also negative for the region mapping the *PTTG1 *locus, although positive for many others. In summary, these data together with those previously reported suggest that primary structural alterations of *PTTG1 *gene are not major mechanisms involved in PTTG1 over-expression.

## Competing interests

The author(s) declares that they have no competing interests.

## Availability and requirements

Genomic sequence from *PTTG1*: 

CpG Island Searcher: 

European Bioinformatic Institute: 

## Authors' contributions

MH, EF and PP participated in experiment design, anatomopathological studies, inclusion criteria and supported the original idea. JJG, AR and RRL performed *in silico *modelling analysis, genome-wide LOH analysis and data interpretation. MAJ, CS and CC were involved in cell culture and RT-qPCRs, western blotting analysis and manuscript preparation. JLR performed MSP analysis, coordinated the project and wrote the manuscript.

## Pre-publication history

The pre-publication history for this paper can be accessed here:



## Supplementary Material

Additional file 1 Sensitivity of methylation-specific PCR. Real time PCR of MSP primer set 2 using serial ratios of converted:untreated DNAs as templates. The graph includes a table showing the cossing point (Ct) attributed to each sample. Even relatively small percentages of methylated DNA can be detected with MSP. Click here for file
