# Supplementary figures and images for: Methylation alterations are not a major cause of PTTG1 missregulation
Source: BMC Cancer. 2008 Apr 21;8:110. doi: 10.1186/1471-2407-8-110 (PMC2377271; doi:10.1186/1471-2407-8-110)

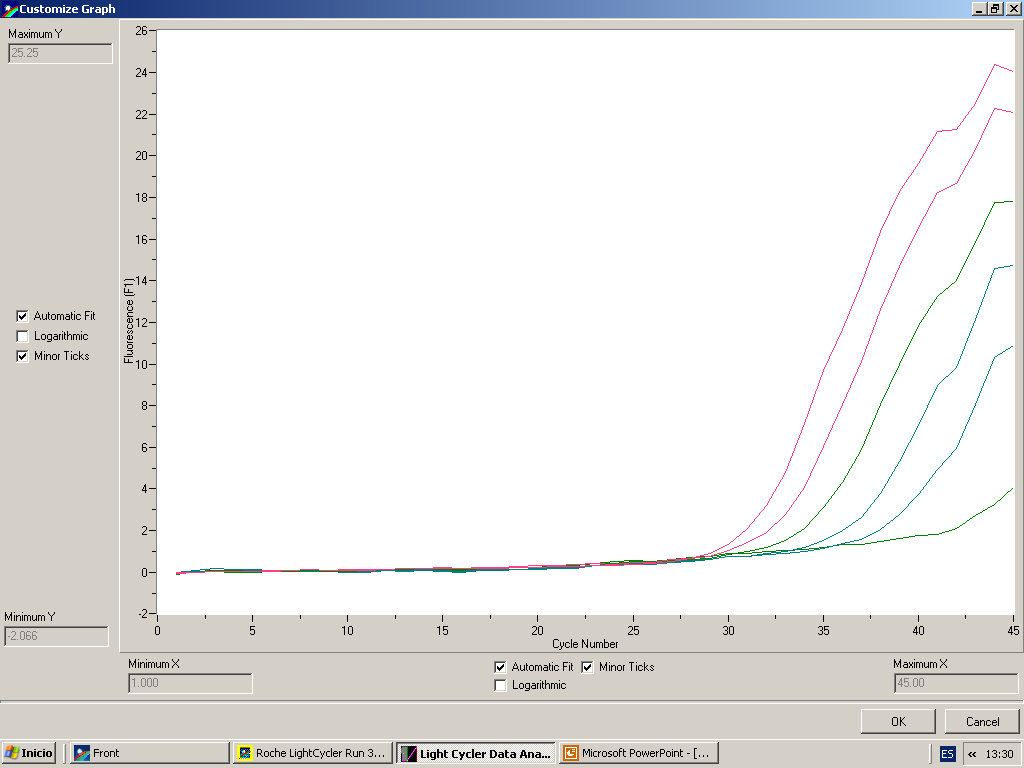


25-

20-

15-

5-

10-

0

5

10

15

20

25

30

35

40

45

Cycle number

Fluorescence (F1)

100:0

90:10

70:30

50:50

30:70

10:90

30,9

32,1

33,8

35,9

38,8

41,7

Ct

Conv:Unconv

**Supplemetary Figure 1**

Supplement: Additional file 1 — Sensitivity of methylation-specific PCR. Real time PCR of MSP primer set 2 using serial ratios of converted:untreated DNAs as templates. The graph includes a table showing the cossing point (Ct) attributed to each sample. Even relatively small percentages of methylated DNA can be detected with MSP. [file 1471-2407-8-110-S1.doc]
